# Supplementary material for: A tetracycline-dependent ribozyme switch allows conditional induction of gene expression in Caenorhabditis elegans
Source: Nat Commun. 2019 Jan 30;10:491. doi: 10.1038/s41467-019-08412-w (PMC6353947; doi:10.1038/s41467-019-08412-w)
Supplement: Supplementary file 1 — Supplementary Information [file 41467_2019_8412_MOESM1_ESM.pdf]

Supplementary Data

A tetracycline-dependent ribozyme switch allows conditional induction of gene expression in *Caenorhabditis elegans*

Lena A. Wurmthaler[1], Monika Sack[1], Karina Gense[2], Jörg S. Hartig[1*], and Martin Gamerdinger[2*]

[1] Department of Chemistry and Konstanz Research School Chemical Biology (KoRS-CB), University of Konstanz, Konstanz, Germany,
[2] Department of Biology and Konstanz Research School Chemical Biology (KoRS-CB), University of Konstanz, Konstanz, Germany

* To whom correspondence should be addressed. Tel: +49-7531-882039; Email: martin.gamerdinger@uni-konstanz.de and Tel: +49-7531-884575; Email: joerg.hartig@uni-konstanz.de

Content:

Supplementary Figure 1
Supplementary Figure 2
Supplementary Figure 3
Supplementary Figure 4
Supplementary Figure 5
Supplementary Figure 6
Supplementary Figure 7
Supplementary Figure 8
Supplementary Figure 9
Supplementary Note 1
Supplementary Note 2
Supplementary Note 3

Supplementary Figure 1. Tetracycline tolerance in *C. elegans*.

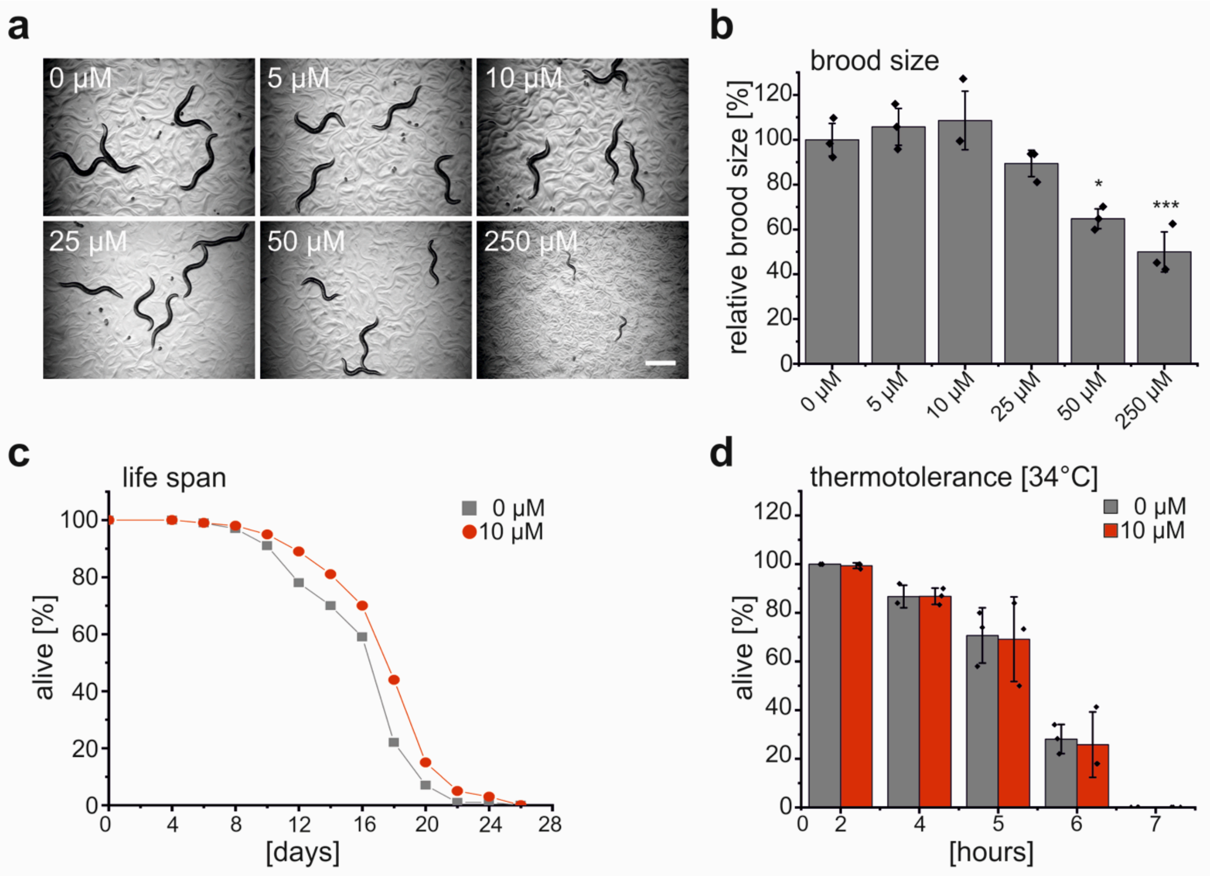

(**a**) Representative images of wild type *C. elegans* N2 worms treated with indicated concentrations of tetracycline from hatch for 3 days at 20°C. Development is only affected at concentrations above 25 µM. A strong developmental delay occurred with 250 µM of tetracycline. Scale bar, 400 µm. (**b**) Bar diagram shows the brood size of N2 worms treated with indicated concentrations of tetracycline from hatch. 30 worms per group were used and experiments were performed in biological triplicates. Error bars, s.d. ***p<0.001, *p<0.05 (two tailed t-test); n=3. (**c**) Life span analysis of N2 worms treated or not with tetracycline (10 µM) from hatch. 100 worms per group were used. Mean life span: 16.49 ± 0.4 days (0 µM) and 17.96 ± 0.38 days (10 µM); n=100. (**d**) Thermotolerance assay of N2 worms treated or not with 10 µM tetracycline from hatch. Day 1 adult animals were heat-shocked at 34°C for the indicated time (2-7 h) and survival rate was determined 16-17 h post-recovery at 20°C. Bar diagram shows the percentage of worms alive. 50 worms per group were used and experiments were performed in biological triplicates. Error bars, s.d. ; n=3. Source data are provided as a Source Data file.

Supplementary Figure 2. Testing of different tetracycline-dependent aptazymes for conditional gene expression in *C. elegans*.

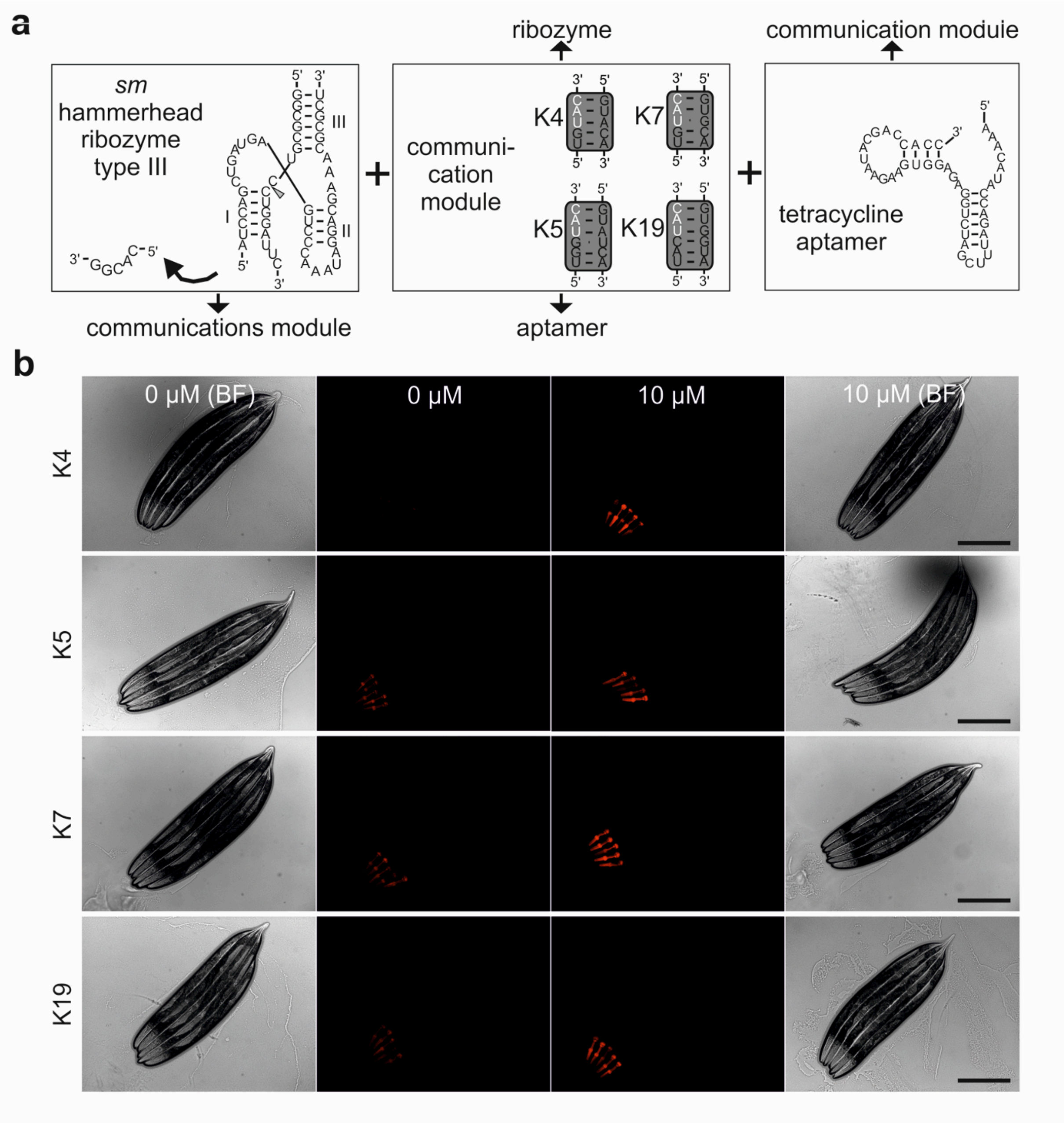

(**a**) Schematic showing the buildup of aptazymes. Stem I of the type III HHR from *Schistosoma mansoni* (sm, left) is opened at indicated position by removing loop region of stem I and connected via different communication modules (K4, K5, K7 or K19; middle) to the tetracycline-binding aptamer (right). See Supplementary Note 1 for further information. (**b**) Microscope images of transgenic worms carrying 3'-UTR aptazyme-regulated *myo-2p::mCherry* reporter with the different communication modules shown in (a). Worms were treated with 10 µM tetracycline from hatch until adulthood (3 days, 20°C). The K4 aptazyme showed best switching performance in *C. elegans*. BF, bright field. Scale bar, 300 µm.

Supplementary Figure 3. Gene induction occurs in various tissues.

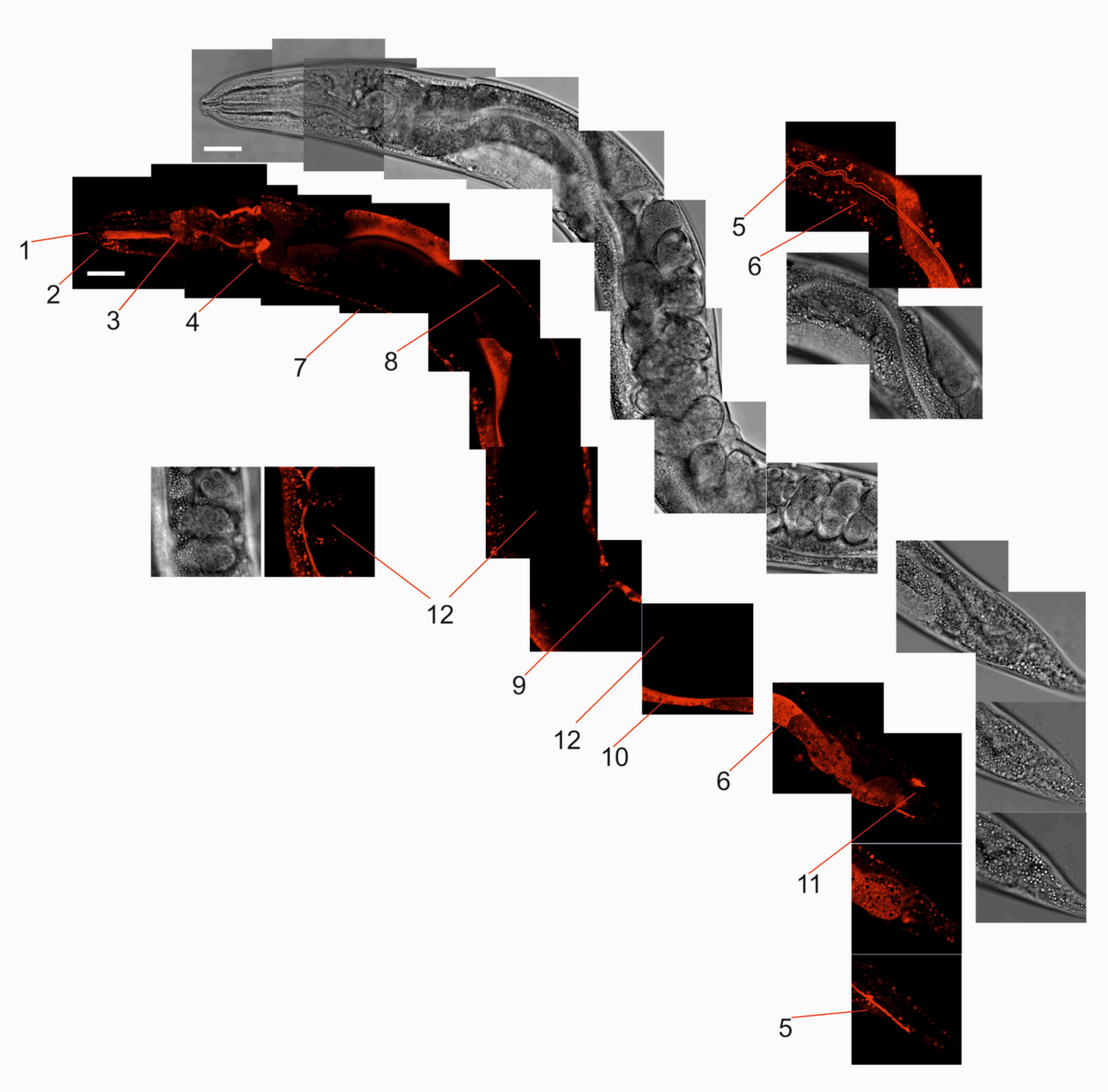

Confocal microscope images of a representative worm carrying the ubiquitous *icd-1p::mCherry* reporter with the tetracycline-regulated aptazyme in the 3'-UTR. The worm was treated with tetracycline (10 µM) from hatch until adulthood (3 days, 20°C). Expression of mCherry was induced in various tissues including 1) mouth tissue, 2) chemosensory neurons, 3) pharynx, 4) anterior nerve ring, 5) excretory system, 6) intestine, 7) ventral nerve cord, 8) dorsal nerve cord, 9) vulva, 10) body wall muscles and 11) lumbar ganglion. Note, that expression lacks in the germline, 12), likely due to general chromatin silencing of the repetitive transgene in this tissue. Bright field images are shown in grey and mCherry fluorescence in red. Scale bar, 30 µm.

Supplementary Figure 4. Catalytically inactive aptazyme.

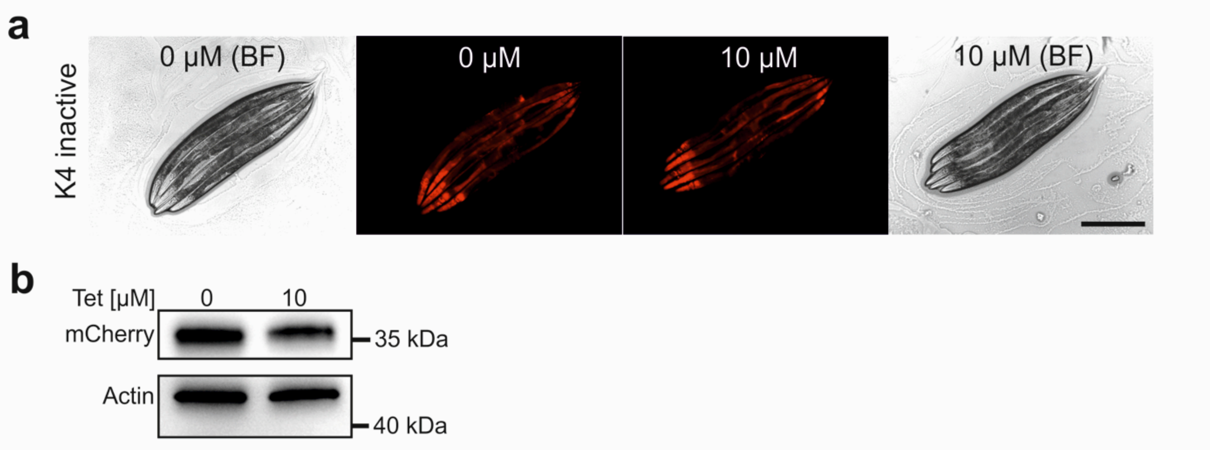

(**a**) Microscope images of transgenic worms carrying the *icd-1p::mCherry* reporter with the mutant, catalytically inactive K4 aptazyme in the 3'-UTR. Worms were treated with 10 μM tetracycline from hatch until adulthood (3 days, 20°C). BF, bright field. Scale bar, 300 μm. (**b**) Immunoblot analysis of mCherry protein levels in animals as shown in (a). Actin served as loading control. Tet, tetracycline.

Supplementary Figure 5. Tetracycline effects on endogenous gene expression.

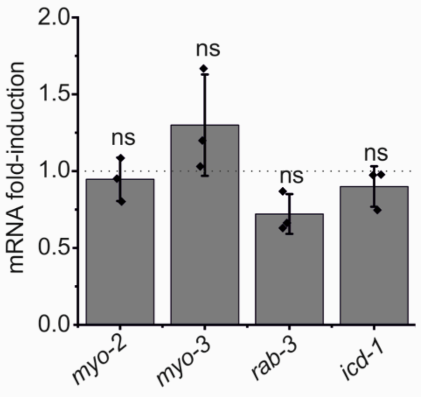

RT-qPCR analysis of indicated transcript levels in N2 worms treated with 10 µM tetracycline from hatch for 3 days at 20°C. Diagram shows the fold-induction of mRNA levels compared to untreated worms. Ama-1 was used as a reference gene. Experiments were performed in biological triplicates. Error bar, s.d. ns, not significant (two-tailed t-test); n=3. Source data are provided as a Source Data file.

Supplementary Figure 6. Worm flow cytometry analysis.

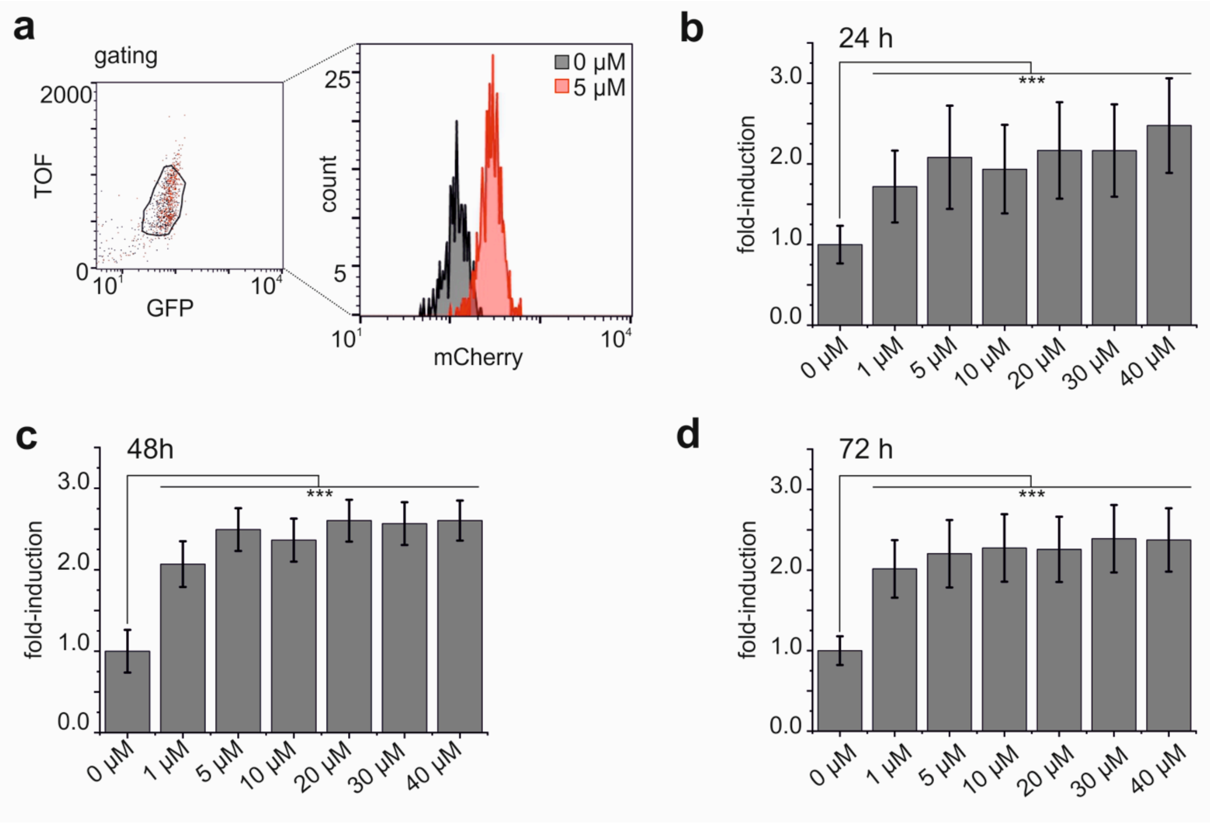

(**a**) Flow cytometry analysis of worms carrying aptazyme-regulated *icd-1p::mCherry* reporter and a constitutively expressed GFP marker (*dpy-30p::GFP*). Synchronized L1 larvae were treated with 5 µM tetracycline for 48 h at 20°C. Left dot plot shows GFP fluorescence intensity against time of flight (TOF) of worms. Marked region shows sorting gate of worms analyzed in the right histogram (relates to Fig. 2d). Right histogram plots mCherry fluorescence intensity against the number of worms. (**b-d**) Extended worm flow cytometry analysis as in (a). Animals were treated with indicated concentrations of tetracycline for 24 h (**b**), 48 h (**c**), and 72 h (**d**) at 20°C. Bar diagrams show the fold-induction of mCherry fluorescence compared to control. Error bars, s.d. ***$p<0.001$ (two tailed t-test); $n>300$. Source data are provided as a Source Data file.

Supplementary Figure 7. RT-qPCR analysis of tetracycline-induced transcript levels.

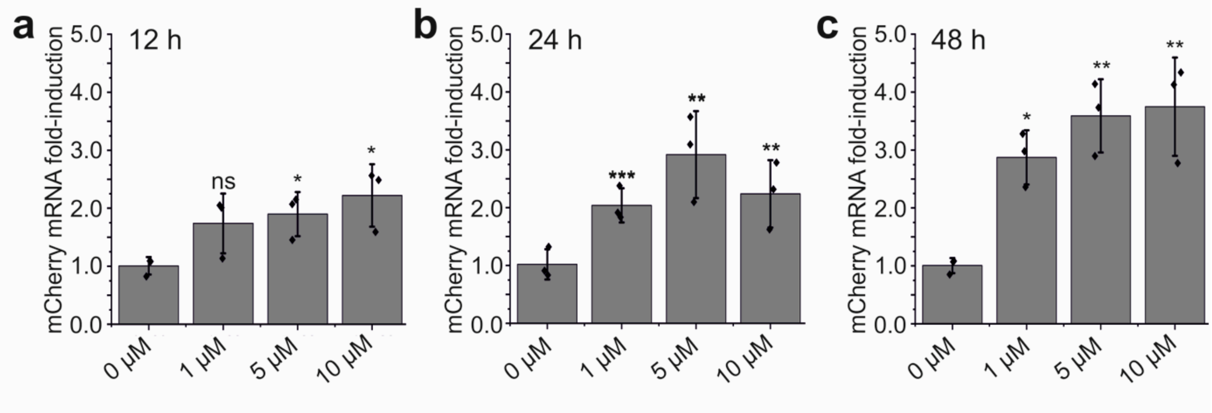

(**a**-**c**) RT-qPCR analysis of mCherry mRNA levels in worms carrying aptazyme-regulated *icd-1p::mCherry* reporter and a constitutively expressed GFP marker (*dpy-30p::GFP*). Synchronized L1 larvae were treated with indicated tetracycline concentrations for (**a**) 12 h, (**b**) 24 h, and (**c**) 48 h at 20°C. GFP was used as a reference gene. Diagrams show the fold-induction of mCherry transcript levels in tetracycline-treated worms compared to non-treated control animals. Experiments were performed in biological triplicates. Error bars, s.d. ***$p<0.001$, **$p<0.01$, *$p<0.05$ (two tailed t-test). ns, not significant; n=3. Source data are provided as a Source Data file.

Supplementary Figure 8. Worm flow cytometry analysis at different developmental stages.

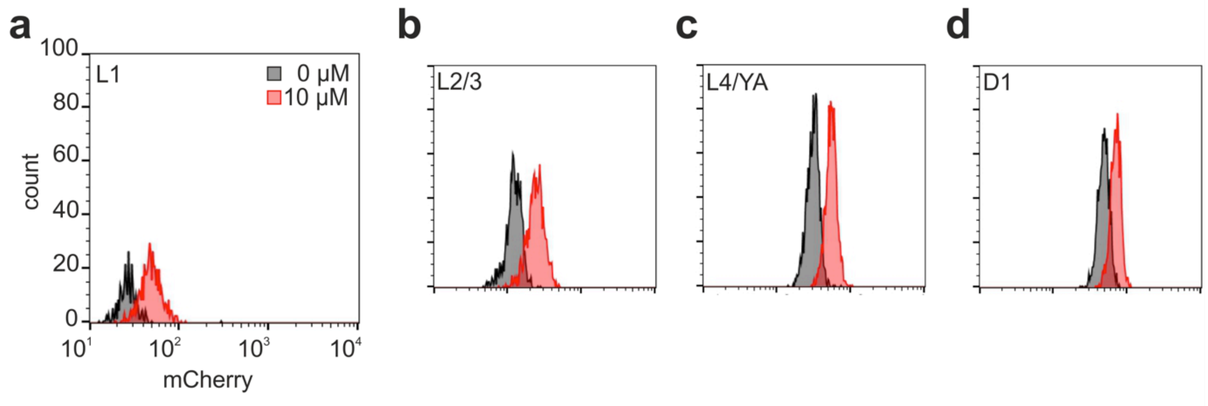

(**a-d**) Histograms of worm flow cytometry analysis shown in main Figure 2e. Worms were treated at indicated developmental stages with 10 μM tetracycline for 24 h at 20°C. Histograms plot mCherry fluorescence intensity against the number of worms. L1-4, larval stage 1-4. YA, young adult. D1, day1 adult.

Supplementary Figure 9. Induced proteotoxic *C. elegans* disease models.

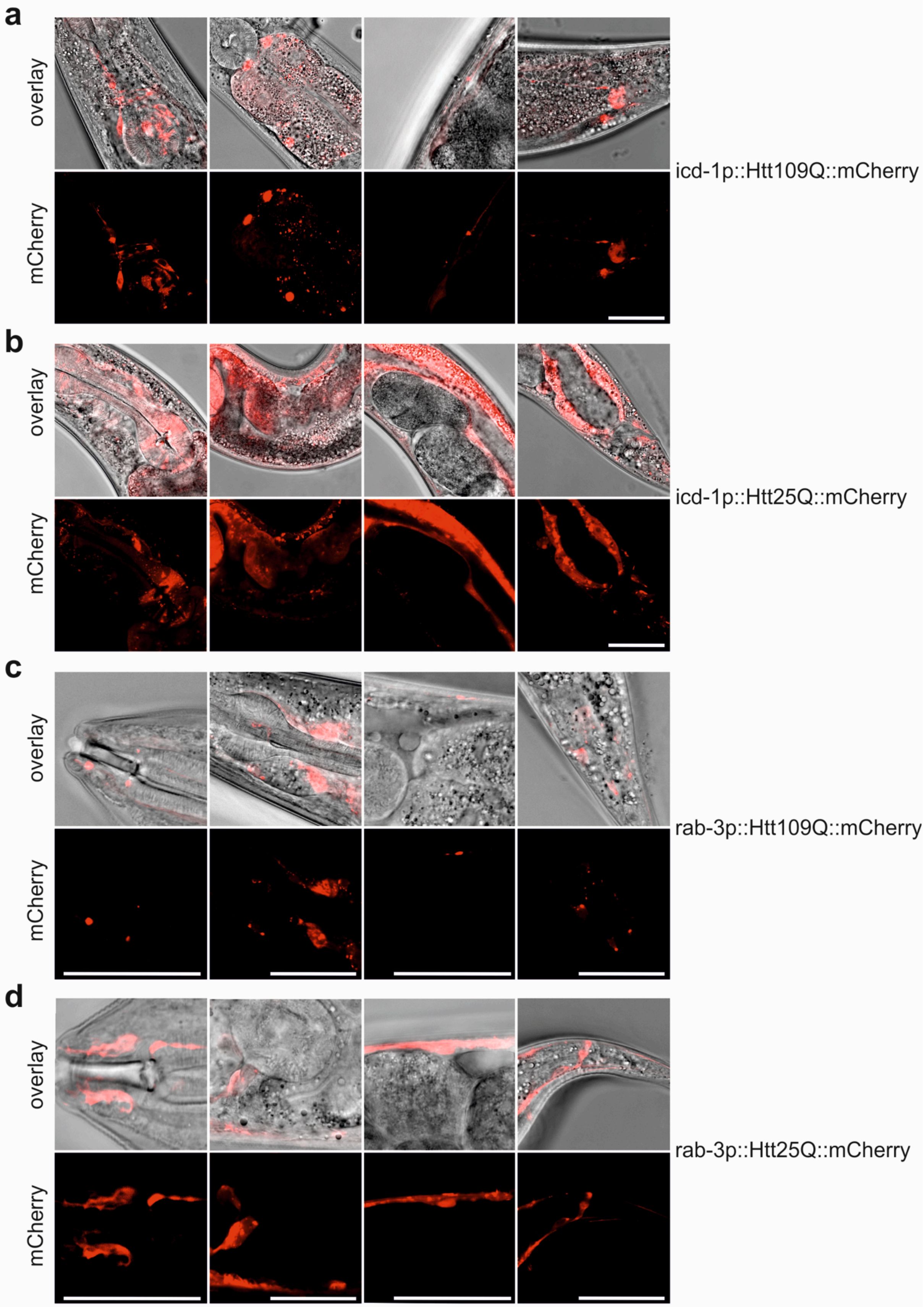

(**a**) Confocal microscope images of ubiquitous, aptazyme-regulated *icd-1p::Htt109Q::mCherry* animals treated with 10 μM tetracycline from hatch for 3 days. Upper row shows the overlay of bright field images with mCherry signal which is shown separated in bottom row. Various tissues are shown containing Htt109Q::mCherry inclusion bodies (pharynx, intestine, body wall muscle and nervous system; from left to right). Scale bar, 30 μm. (**b**) Same analysis as in (a) but with *icd-1p::Htt25Q::mCherry* animals. (**c**) Confocal microscope images of worms carrying pan-neuronal, aptazyme-regulated *rab-3p::Htt109Q::mCherry* construct. Various neurons are shown containing Htt109Q::mCherry inclusion bodies (chemosensory neurons, nerve ring, ventral nerve cord, tail ganglion; from left to right). Scale bar, 30 μm. (**d**) Same analysis as in (b) but with *rab-3p::Htt25Q::mCherry* animals.

Supplementary Note 1. Ribozyme sequences and constructs.

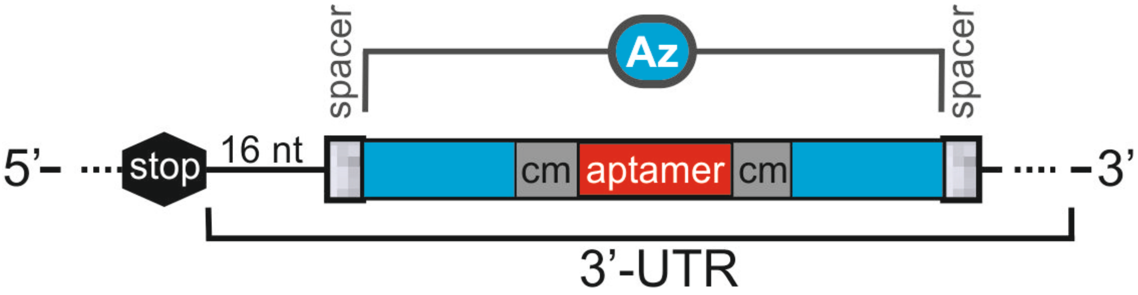

**DNA sequences 5' to 3'**	Ribozyme sequence, communication module, aptamer domain,<br>**A-to-G inactivation site**
**HHR type 3 active (Rz)**	GGCGCGTCCTGGATTCCACGGTACATCCAGCTGATGAGTCCCAAATAGGACGA**A**ACGCGCT
**HHR type 3 inactive (Rz)**	GGCGCGTCCTGGATTCCACGGTACATCCAGCTGATGAGTCCCAAATAGGACGA**G**ACGCGCT
**K4 active aptazyme (Az)**	GGCGCGTCCTGGATTCGTACAAAACATACCAGATTTCGATCTGGAGAGGTGAAGAATACGACCA<br>CC TGTACATCCAGCTGATGAGTCCCAAATAGGACGA**A**ACGCGCT
**K4 inactive aptazyme (Az)**	GGCGCGTCCTGGATTCGTACAAAACATACCAGATTTCGATCTGGAGAGGTGAAGAATACGACCA<br>CC TGTACATCCAGCTGATGAGTCCCAAATAGGACGA**G**ACGCGCT
**K5 aptazyme (Az)**	GGCGCGTCCTGGATTCGTATCAAAACATACCAGATTTCGATCTGGAGAGGTGAAGAATACGACC<br>ACCTGGTACATCCAGCTGATGAGTCCCAAATAGGACGA**A**ACGCGCT
**K7 aptazyme (Az)**	GGCGCGTCCTGGATTCGTGCAAAACATACCAGATTTCGATCTGGAGAGGTGAAGAATACGACCA<br>CC TGTACATCCAGCTGATGAGTCCCAAATAGGACGA**A**ACGCGCT
**K19 aptazyme (Az)**	GGCGCGTCCTGGATTCGTGGTAAAACATACCAGATTTCGATCTGGAGAGGTGAAGAATACGACC<br>ACCTACTACATCCAGCTGATGAGTCCCAAATAGGACGA**A**ACGCGCT

**DNA sequences 5' to 3'**	3'-UTRs with aptazyme (**Az**) sequence, insulated by 5' and 3' **CAAA-**spacers, 16 nt downstream of STOP codon (***TAG/TAA***)
unc-54 **3'-UTR**	***TAG***TACCCAGCTTTCTTGT**CAAACAAACAAA**-Az -**CAAACAAACAAA**ACAAAGTGGGTGATATC<br>TGAGCTCCGCATCGGCCGCTGTCATCAGATCGCCATCTCGCGCCCGTGCCTCTGACTTCTAAGTCC<br>AATTACTCTTCAACATCCCTACATGCTCTTTCTCCCTGTGCTCCCACCCCCTATTTTTGTTATTAT<br>CAAAAAACTTCTCTTAATTTCTTTGTTTTTTAGCTTCTTTTAAGTCACCTCTAACAATGAAATTG<br>TGTAGATTCAAAAATAGAATTAATTCGTAATAAAAAGTCGAAAAAAATTGTGCTCCCTCCCCCC<br>ATTAATAATAATTCTATCCCAAAATCTACACAATGTTCTGTGTACACTTCTTATGTTTTTTACTT<br>CTGATAAATTTTTTTGAAACATCATAGAAAAAACCGCACACAAAATACCTTATCATATGTTACG<br>TTTCAGTTTATGACCGCAATTTTTATTTCTTCGCACGTCTGGGCCTCTCATGACGTCAAATCATG<br>CTCATCGTGAAAAAGTTTTGGAGTATTTTTGGAATTTTTCAATCAAGTGAAAGTTATGAAATTA<br>ATTTTCCTGCTTTTGCTTTTTGGGGTTTCCCCTATTGTTTGTCAAGATTTCGAGGACGGCGTTTT<br>TCTTGCTAAAATCACAAGTATTGATGAGCACGATGCAAGAAAGATCGGAAGAAGGTTTGGGTTT<br>GAGGCTCAGTGGAAGGTGAGTAGAAGTTGATAATTTGAAAGTGGAGTAGTGTCTATGGGGTTTT<br>TGCCTTAAATGACAGAATACATTCCCAATATACCAAACATAACTGTTT
icd-1 **3'-UTR**	***TAG***GATCTTCGAACCTATC**CAAACAAACAAA**-Az-**CAAACAAACAAA**ACTACCCTTTCATCGACC<br>AATTCGCGAGTTTTGTGAGATGAACGGTACGGATAGCATCCGCATTAGTAATCCCATTTTTCTTG<br>TTACTGTTGATCGATTTCAATGAATCTCTTTAAAAAAATACTTGCTCTGTCACCTTTTTTTCCGC<br>CCCCATTAGTGTTCGTTGTTATACTTGTTTTGTATACAAATTTATTCCCGATCCTCAAATGGCTA<br>ATAAATGTTAGCTATGTATTTAAAGCAAGCTTTCTACGTTCCGATGATTTTGAATTCTTAGGGG<br>TTCCGGGGATCCTTCTAGTAATATCTGTTCCTCGGTGTTTATTTTCCGTTATTTTGGGTAGTTTT<br>CCACTGACTTCCATTTTGTTAAACTCAATAAAAGTTGCACAAATA
unc-119 **3'-UTR**	***TAA***ATATTTAATACAAAAA**CAAACAAACAAA**-Az-**CAAACAAACAAA**TGTTCTGGATAA<br>TTATTCTGTCGAATAGAAAAAAAACTCCAAATGTGATTAAATTCCAATAATTCCTGTCTAGTTT<br>GTTCCTTCCCCTTCCCCTTCTCATGTTCAATGCATTCCTAAGCTTTTCAGTTCCCCCCTTGTTTCT<br>ATATTTTTTCGCTGTCCTGTCACACTCGCTAAAAACACTAATCACACGGAAATCTGTTTTCAATA<br>AAAACTCCAACTTTAACTCATTTTCAATTTCA

Supplementary Note 2. *C. elegans* strains.

DEU101 *gamEx1* [*myo-2p::mCherry::unc-54 3-UTR-HHR type 3 active* (2.5 ng/µl), *dpy-30p::GFP* (10 ng/µl), DNA ladder (90 ng/µl)]

DEU102 *gamEx2* [*myo-2p::mCherry::unc-54 3-UTR-HHR type 3 inactive* (2.5 ng/µl), *dpy-30p::GFP* (10 ng/µl), DNA ladder (90 ng/µl)]

DEU103 *gamEx3* [*myo-2p::mCherry::unc-54 3-UTR-K4 HHR type 3 active* (2.5 ng/µl), *dpy-30p::GFP* (10 ng/µl), DNA ladder (90 ng/µl)]

DEU104 *gamEx4* [*myo-2p::mCherry::unc-54 3-UTR-K5 HHR type 3 active* (2.5 ng/µl), *dpy-30p::GFP* (10 ng/µl), DNA ladder (90 ng/µl)]

DEU105 *gamEx5* [*myo-2p::mCherry::unc-54 3-UTR-K7 HHR type 3 active* (2.5 ng/µl), *dpy-30p::GFP* (10 ng/µl), DNA ladder (90 ng/µl)]

DEU106 *gamEx6* [*myo-2p::mCherry::unc-54 3-UTR-K19 HHR type 3 active* (2.5 ng/µl), *dpy-30p::GFP* (10 ng/µl), DNA ladder (90 ng/µl)]

DEU107 *gamIs7* [*icd-1p::mCherry::icd-1 3-UTR-K4 HHR type 3 active* (2.5 ng/µl), *dpy-30p::GFP* (10 ng/µl), DNA ladder (90 ng/µl)]

DEU108 *gamEx8* [*myo-3p::mCherry::unc-54 3-UTR-K4 HHR type 3 active* (2.5 ng/µl), *dpy-30p::GFP* (10 ng/µl), DNA ladder (90 ng/µl)]

DEU109 *gamEx9* [*rab-3p::mCherry::unc-54 3-UTR-K4 HHR type 3 active* (2.5 ng/µl), *dpy-30p::GFP* (10 ng/µl), DNA ladder (90 ng/µl)]

DEU110 *gamIs10* [*icd-1p::Htt25Q::mCherry::icd-1 3-UTR-K4 HHR type 3 active* (50 ng/µl), *dpy-30p::GFP* (10 ng/µl), DNA ladder (20 ng/µl)]

DEU111 *gamEx11* [*icd-1p::Htt109Q::mCherry::icd-1 3-UTR-K4 HHR type 3 active* (50 ng/µl), *dpy-30p::GFP* (10 ng/µl), DNA ladder (20 ng/µl)]

DEU112 *gamEx12* [*rab-3p::Htt25Q::mCherry::unc-54 3-UTR-K4 HHR type 3 active* (50 ng/µl), *dpy-30p::GFP* (10 ng/µl), DNA ladder (20 ng/µl)]

DEU113 *gamEx13* [*rab-3p::Htt109Q::mCherry::unc-54 3-UTR-K4 HHR type 3 active* (50 ng/µl), *dpy-30p::GFP* (10 ng/µl), DNA ladder (20 ng/µl)]

DEU114 *gamEx14* [*icd-1p::mCherry::icd-1 3-UTR-K4 HHR type 3 inactive* (2.5 ng/µl), *dpy-30p::GFP* (10 ng/µl), DNA ladder (90 ng/µl)]

DEU115 *unc-119(ed3)III*; *gamEx15* [*unc-119p::Cbr-unc-119::unc-119 3-UTR* (10 ng/µl), *eft-3p::GFP::H2B* (10 ng/µl), DNA ladder (90 ng/µl)]

DEU116 *unc-119(ed3)III*; *gamEx16* [*unc-119p::Cbr-unc-119::unc-119 3-UTR-K4 HHR type 3 active* (10 ng/µl), *eft-3p::GFP::H2B* (10 ng/µl), DNA ladder (90 ng/µl)]

Supplementary Note 3. QPCR primer.

mCherry-F<br>mCherry-R	5'-ATTACGATGCTGAGGTGAAGAC-3'<br>5'-CGATAGTGTAATCCTCGTTGTG-3'
GFP-F<br>GFP-R	5'-CTACCTGTTCCATGGCCAACAC-3'<br>5'-ATAACCTTCGGGCATGGCACTC-3'
Myo-2-F<br>Myo-2-R	5'-GAAAGAGCCGCTAAGTGC-3'<br>5'-CCCTTGTTCACCCATTCG-3'
Myo-3-F<br>Myo-3-R	5'-GAAGCCAAAGCCACCAAAG-3'<br>5'-GCGTTGTAACGGACAGTTC-3'
Rab-3-F<br>Rab-3-R	5'-ACTGTGTTCCGTGGAGAC-3'<br>5'-TAGTAGGCGGTGGTGATG-3'
Icd-1-F<br>Icd-1-R	5'-TGCTCCCAGGAATCCTCAAC-3'<br>5'-TGGCACATCTTCGTCCTCTC-3'
Ama-1-F<br>Ama-1-R	5'-AGCCAGGAACTTCGGCTCAG-3'<br>5'-CATAAGTCGGCGAGCTTG-3'
